# Antibiofilm and Antihemolytic Activities of *Actinostemma lobatum* Extract Rich in Quercetin against *Staphylococcus aureus*

**DOI:** 10.3390/pharmaceutics16081075

**Published:** 2024-08-16

**Authors:** Jin-Hyung Lee, Yong-Guy Kim, Ji-Su Choi, Yong Tae Jeong, Buyng Su Hwang, Jintae Lee

**Affiliations:** 1 School of Chemical Engineering, Yeungnam University, 280 Daehak-Ro, Gyeongsan 38541, Republic of Korea; jinhlee@ynu.ac.kr (J.-H.L.); yongguy7@ynu.ac.kr (Y.-G.K.); 2 Nakdonggang National Institute of Biological Resources, 137, Donam 2-gil, Sangju-si 37242, Republic of Korea; ccc6112@nnibr.re.kr (J.-S.C.); ytjeong@nnibr.re.kr (Y.T.J.)

**Keywords:** *Actinostemma lobatum*, antibiofilm, antihemolysis, quercetin, *Staphylococcus aureus*

## Abstract

*Staphylococcus aureus* biofilm formation is a pivotal mechanism in the development of drug resistance, conferring resilience against conventional antibiotics. This study investigates the inhibitory effects of *Actinostemma lobatum* (*A. lobatum*) Maxim extracts on *S. aureus* biofilm formation and their antihemolytic activities, with a particular focus on identifying the active antibiofilm and antihemolysis compound, quercetin. Seven solvent extracts and twelve sub-fractions were evaluated against four *S. aureus* strains. The ethyl acetate fraction (10 to 100 μg/mL) significantly hindered biofilm formation by both methicillin-sensitive and -resistant strains. Bioassay-guided isolation of the ethyl acetate extract identified quercetin as the major antibiofilm compound. The ethyl acetate extract was found to contain 391 μg/mg of quercetin and 30 μg/mg of kaempferol. Additionally, the *A. lobatum* extract exhibited antihemolytic activity attributable to the presence of quercetin. The findings suggest that quercetin-rich extracts from *A. lobatum* and other quercetin-rich foods and plants hold promise for inhibiting resilient *S. aureus* biofilm formation and attenuating its virulence.

## 1. Introduction

*Staphylococcus aureus* is a Gram-positive bacterium that naturally inhabits human skin and the upper respiratory tract, posing a risk for skin infections, respiratory issues, and foodborne illnesses. The opportunistic pathogen often acquires its antibiotic resistance during antibiotic treatment and is responsible for hospital-acquired infections [1]. Consequently, methicillin-resistant *S. aureus* (MRSA) strains and vancomycin-resistant *S. aureus* strains (VRSA) are worldwide threats. Additionally, *S. aureus* secretes an array of toxins, including hemolysins, enterotoxins, and antigenic and cytolytic toxins, which contribute to its virulence [2]. Of the three hemolysins, α-hemolysin, produced by the *hla* gene, has been the focus of extensive research as a virulence factor in *S. aureus*. Numerous strategies have been explored to counteract the α-hemolysin toxin [2]. Moreover, the biofilm formation of *S. aureus* on host cells, indwelling medical devices, food processing surfaces, and implants significantly increases its drug tolerance due to biofilm structures’ inherent protection from physical and chemical stresses [3]. Hence, various methods to inhibit or disperse biofilms have been explored [4,5,6]. Our ongoing research aims to identify novel compounds that not only inhibit biofilm formation but also possess antihemolytic properties, specifically targeting *S. aureus*. This quest has led to the investigation of phytochemicals for their potential therapeutic benefits. For instance, previous studies have investigated the antibiofilm activities of 498 plant extracts [7] and 83 plant essential oils [8] against *S. aureus*, highlighting the vast potential of plant-based compounds in combating this pathogen. Additionally, phytopigment alizarin and a plant extract from *Iris pallida* have been shown to inhibit multispecies biofilm development [9,10].

In this study, we explored the extracts of *Actinostemma lobatum* Maxim for its potential to inhibit biofilm formation and hemolytic activity in *S. aureus*. Seven solvent extracts and twelve sub-fractions of *A. lobatum* aerial parts were obtained and subjected to testing. *A. lobatum* is a semi-aquatic genus of flowering plants in the family Cucurbitaceae, indigenous to East Asia [11,12]. Previous studies have highlighted various beneficial properties of *A. lobatum* extract, including antioxidant [13], antitumor [14,15], antithrombotic [16], and antifungal activities [17]. Also, the *A. lobatum* Maxim kernel is rich in essential fatty acids and proteins [18]. The active compound responsible for antibiofilm activity was identified and quantified with bioassay-guided fractionation, HPLC, and NMR analyses. The efficacy of biofilm inhibition was confirmed by a crystal violet staining assay, live imaging microscopy, and scanning electron microscopy. Additionally, the potential of the active fraction to prevent hemolysis of sheep red blood cells by *S. aureus* was assessed, offering insights into its therapeutic capabilities against virulence mechanisms beyond biofilm formation.

## 2. Materials and Methods

### 2.1. Preparation of Plant Materials

**Figure 1:**
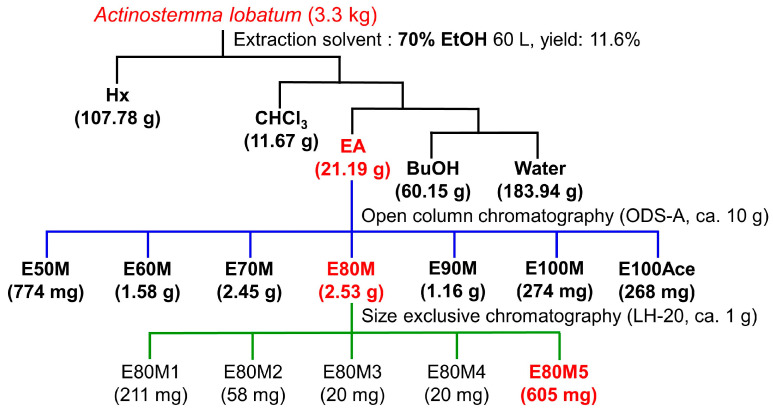
Schematic diagram for fractionation of *A. lobatum* extract.

An *Actinostemma lobatum* specimen was collected in Gongju-si, Chungcheongnam-do, Korea, in August 2021. A voucher specimen (NNIBRVP93410) was deposited in the Library of Nakdonggang National Institute of Biological Resources (Sangju-si, Republic of Korea). An ethanolic extract of the plant was prepared by macerating 3.3 kg of the aerial parts and successively extracting with 70% ethanol (2 × 30 L) at room temperature. The resulting mixture was filtered, and the filtrate was completely evaporated under vacuum to yield a 70% EtOH extract. The crude extract (390 g, yield to 11.8%) was re-suspended in water and partitioned successively with hexane (Hx, 107.78 g), chloroform (C, 11.67 g), ethyl acetate (EA, 21.19 g), *n*-butanol (BuOH, 60.51 g), and water (183.94 g) (Figure 1).

The active EA fraction (ca. 10 g) was collected and subjected to chromatography on a reversed-phase silica column (RP-18, 40–63 μm, LiChroprep^®^, Darmstadt, Germany). Elution was performed using mixtures of MeOH and H_2_O or 100% acetone, with decreasing polarity (MeOH:H_2_O = 5:5; 6:4; 7:3; 8:2; 9:1; 10:0) to give seven fractions (E50M-E100Ace). The active fraction (E80M) was further chromatographed on a Sephadex LH-20 open column and eluted with 100% MeOH to yield five sub-fractions (E80M1-E80M5). Subsequent biological activity evaluation confirmed the efficacy of the re-fraction E80M5.

The E80M5 sub-fraction underwent purification via prep-HPLC, using eluents water (A) and MeCN (B), each containing 0.1% formic acid, at a flow rate of 15 mL/min. The process utilized a gradient solvent system, starting at 10% B for 5 min, ramping up to 50% B over the next 45 min, and concluding at 95% B for the final 5 min. A 320 nm UV detector was employed to detect the presence of quercetin and kaempferol for subsequent analysis by NMR. Quercetin and kaempferol were obtained from Sigma-Aldrich (St. Louis, MO, USA).

### 2.2. Bacterial Strains and Cell Growth Measurements

This research utilized two methicillin-sensitive *S. aureus* strains (MSSA; ATCC 6538 and ATCC 25923) and two methicillin-resistant *S. aureus* strains (MRSA; MW2 and ATCC 33591), all sourced from the American Type Culture Collection (Manassas, VA, USA). Experiments involving the ATCC 6538 and ATCC 25923 strains utilized Luria–Bertani (LB) broth, whereas experiments with the ATCC 33591 and MW2 strains were performed using LB liquid broth with 0.2% glucose, with all conditions maintained at 37 °C. For planktonic cell growth assessments, optical densities were measured at 600 nm (OD_600_) using a Multiskan SkyHigh Photometer (Thermo Fisher Scientific, Waltham, MA, USA). The MIC was determined as the minimum concentration that inhibited cell growth. MICs were determined following the CLSI methods [19]. In brief, a 0.5 McFarland suspension of *S. aureus* cells was initially diluted 1:150, and then further diluted 1:2 in fresh LB medium. The mixture was then dispensed into a 96-well plate and incubated for 24 h at 37 °C without agitation.

### 2.3. Analyses of Quercetin Using HPLC and NMR

Quercetin concentrations were analyzed using reverse-phase HPLC on a 4.6 × 250 mm ZORBAX Eclipse XDB-C18 column (Agilent Technology, Santa Clara, CA, USA). The mobile phases, water (A) and MeCN (B), both contained 0.1% formic acid, and the flow rate was set at 1 mL/min. The gradient solvent system started at 5% B for 5 min, increased to 95% B over 35 min, and held at 95% B for an additional 5 min, monitored by a 360 nm UV detector. Plant extracts, along with commercial quercetin and kaempferol standards, were dissolved in methanol and filtered through a 0.2 μm syringe filter. Quercetin’s chromatographic peak was identified by comparing retention times and UV/visible spectra with the standards.

Nuclear magnetic resonance (NMR) spectra were obtained on a Varian VNMRS 500 NMR spectrometer (Varian, Palo Alto, CA, USA) operating at frequencies of 500 MHz for ^1^H and 125 MHz for ^13^C. Chemical shifts in the proton and carbon spectra were measured in methanol-*d*4 solution and referenced to residual solvent peaks at 3.3 ppm and 49.0 ppm. Additionally, the NMR spectra for quercetin and kaempferol were cross-referenced with a prior report [20].

### 2.4. Crystal Violet Biofilm Assay

A crystal violet staining assay was performed in 96-well plates following the protocol outlined as previously reported [19]. *S. aureus* cells (~10^7^ CFU/mL) were inoculated into LB medium and fractions of plant extracts were added to the wells of the 96-well plates. The plates were incubated for 24 h at 37 °C without shaking. Afterward, biofilm formation was assessed by discarding planktonic cells and washing the plates three times using distilled water. Subsequently, the biofilm cells were stained using a 0.1% crystal violet solution (300 μL) for 20 min. Following staining, the plates were washed three times with water to remove any excess dye. Next, the crystal-violet-stained biofilm cells were extracted with 300 μL of 95% ethanol through vigorous shaking. Absorbance readings were taken at 570 nm (OD_570_) using a Multiskan SkyHigh Photometer (Thermo Fisher Scientific, Waltham, MA, USA). Results regarding biofilm formation were derived from three independent cultures, each with six replicate wells.

### 2.5. Microscopic Analysis of Biofilms

After cultivating *S. aureus* biofilms in 96-well plates with or without plant extract (0, 20, 50, and 100 μg/mL) for 24 h at 37 °C, planktonic cells were removed by washing the wells three times with distilled water. The remaining live biofilm cells were visualized using the iRiS™ Digital Cell Imaging System (Logos Biosystems, Anyang, Republic of Korea). Color-coded 3D images of the biofilms were then generated using ImageJ 1.53k software. Additionally, scanning electron microscopy (SEM) was employed to observe the impact of the plant extract on biofilm reduction, following the methodology described as previously reported [21]. For the SEM analysis, *S. aureus* ATCC 6538 cells (~10^7^ CFU/mL) were introduced into 1 mL of fresh LB medium, with or without plant extract (0, 10, and 50 μg/mL) and quercetin (5 and 20 μg/mL), in a 96-well plate. A piece of nylon membrane (~0.16 cm^2^) was placed in each well, and the *S. aureus* cells were cultured for 24 h at 37 °C without agitation. The biofilms formed on the membranes were fixed using a solution that comprised 2.5% glutaraldehyde and 2% formaldehyde for 24 h, and dehydrated using ethanol. Following dehydration, the biofilms were dried using a critical-point dryer (HCP-2, Hitachi, Tokyo, Japan), coated with a Precision Etching Coating System (Gatan, Inc., Pleasanton, CA, USA), and examined under a field emission scanning electron microscope S-4800 (Hitachi, Tokyo, Japan).

### 2.6. Hemolytic Activity Assay

The hemolytic activity on defibrinated sheep blood (Cat No. MB-S1876, MBcell, Seoul, Republic of Korea) was assessed following the protocol outlined as previously reported [7]. *S. aureus* ATCC 6538 cells (~2 × 10^7^ CFU/mL) were inoculated in 2 mL of fresh LB medium and cultured with the EA extract (0, 2, 5, 10, 20, and 50 μg/mL) and standard quercetin (0, 1, 2, 5, 10, and 20 μg/mL) for 24 h at 37 °C, with agitation at 250 rpm. Sheep red blood cells were collected by centrifugation at 3000× *g* for 2 min, then washed three times with PBS to eliminate any remaining components. They were then carefully re-suspended in a 3.3% PBS buffer. Following this, 100 μL of the *S. aureus* culture was mixed with 1 mL of the red blood cell suspension and incubated for 1 h at 37 °C, shaking at 250 rpm. As negative controls, LB medium and PBS were used separately. After the incubation, the mixtures were centrifuged at 16,600× *g* for 10 min, and the optical densities of the supernatants were measured at 543 nm.

### 2.7. Statistical Analysis

The data obtained were subjected to statistical analysis using one-way ANOVA followed by Dunnett’s test in SPSS version 23 (SPSS Inc., Chicago, IL, USA). The results are expressed as means and standard deviations, with significance set at *p* values < 0.05.

## 3. Results

### 3.1. Antibiofilm Activity of A. lobatum Extract

**Figure 2:**
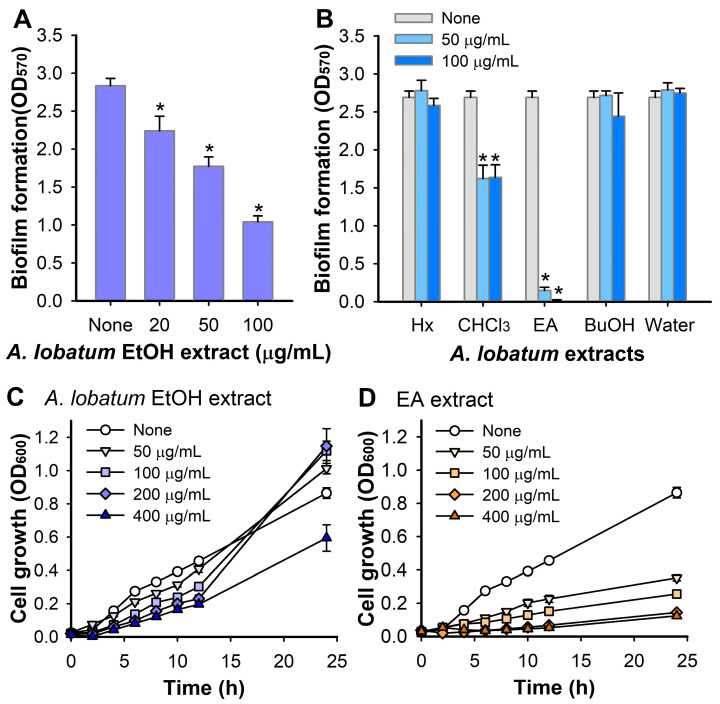
Biofilm and planktonic growth inhibition by *A. lobatum* extract. Biofilm formation (OD_570_) of *S. aureus* ATCC 6538 was measured in the presence of *A. lobatum* EtOH extract (**A**) and its solvent extract (**B**) after 24 h in 96-well plates. Planktonic cell growth of *S. aureus* ATCC 6538 in the presence of EtOH extract (**C**) or EA extract (**D**) was measured at 600 nm in 96-well plates. *, *p* < 0.05 vs. non-treated controls (None).

As shown in Figure 1, the ethanol (EtOH) extract of the aerial parts of *A. lobatum* was obtained and the antibiofilm activity of the EtOH extract was initially tested against *S. aureus* in 96-well plates. The extract inhibited *S. aureus* biofilm formation in a dose-dependent manner (Figure 2A). Specifically, the extract at 50 and 100 μg/mL reduced biofilm formation by 37% and 63%, respectively. Also, the antimicrobial activity of the EtOH extract of *A. lobatum* was measured, and at 50–400 μg/mL, at a concentration of 100 μg/mL, it modestly postponed the growth of planktonic cells, with an MIC exceeding 400 μg/mL (Figure 2C). These findings suggest that the antibiofilm properties of the *A. lobatum* extract primarily stem from its capacity to prevent biofilm formation, rather than from its capacity to inhibit the growth of planktonic cells or cause cell death.

### 3.2. Bioassay-Guided Fractionation and Isolation

**Figure 3:**
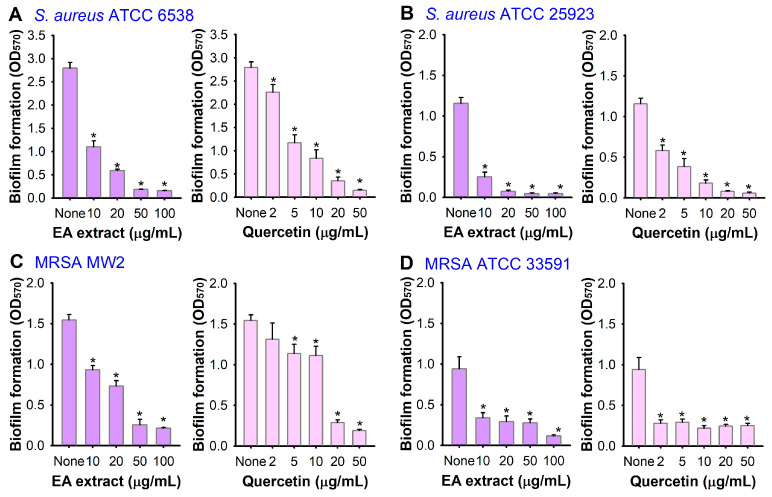
Biofilm inhibition by *A. lobatum* EA extract and quercetin. Biofilm formation (OD_570_) of *S. aureus* ATCC 6538 (**A**), ATCC 25923 (**B**), MRSA MW2 (**C**) and MRSA ATCC 33591 (**D**) was measured in the presence of EA extract after 24 h in 96-well plates. *, *p* < 0.05 vs. non-treated controls (None).

Five different solvents, viz. hexane (Hx), chloroform (CHCl_3_), ethyl acetate (EA), butanol (BuOH), and water, were used to further extract the crude extract, and the fractionation amounts are shown in Figure 1. All extracts at 50 or 100 μg/mL were tested for antibiofilm activity against *S. aureus* ATCC 6538. Among the five extracts, the EA extract was most effective at inhibiting *S. aureus* biofilm formation, while the CHCl_3_ extract showed a minor effect on the biofilm formation and the other extracts had no effect (Figure 2B). Particularly, the EA extract at a concentration of 50 μg/mL diminished biofilm formation by over 95%, whereas it modestly affected the growth of planktonic cells with an MIC exceeding 400 μg/mL (Figure 2D). A more detailed biofilm assay also confirmed that the EA extract dose-dependently inhibited biofilm formation, as 20 μg/mL of EA extract inhibited biofilm formation by 79% (Figure 3A).

In addition, the EA extract effectively inhibited the biofilm formation of MSSA ATCC 25293, MRSA MW2, and MRSA ATCC 33591 (Figure 3B–D). In particular, the EA extract dose-dependently inhibited the biofilm formation of two MRSA strains. Specifically, the EA extract at 50 μg/mL reduced the biofilm formation of MRSA MW2 and MRSA ATCC 33591 by more than 70%.

**Figure 4:**
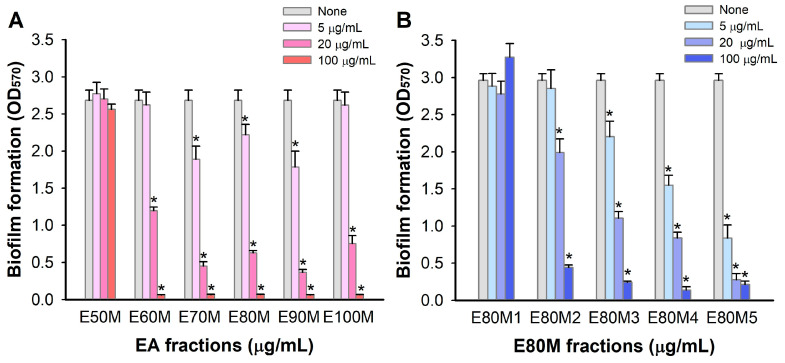
Biofilm inhibition by sub-EA fractions. Biofilm formation (OD_570_) of *S. aureus* ATCC 6538 was quantified in the presence of six sub-EA fractions (**A**) and six E80M fractions (**B**) after 24 h culture. *, *p* < 0.05 vs. non-treated controls (None).

Further fractionation of the EA extract was performed with a reversed-phase silica column using mixtures of MeOH and H_2_O or 100% acetone, and seven gradient fractions were obtained as shown in Figure 1. The fractions at 5, 20, and 100 μg/mL were again tested for antibiofilm activity, and most fractions, except E50M1, were active. Notably, three fractions (E70M, E80M, and E90M) at 20 and 100 μg/mL were very active, as they inhibited biofilm formation more than 76% (Figure 4A). Hence, the most abundant and active E80M was further chromatographed to yield five sub-fractions (E80M1-E80M5). Among five fractions, E80M5 was most active (Figure 4B). For example, E80M5 at 5 and 20 μg/mL reduced *S. aureus* biofilm formation by more than 70% and 90%, respectively.

### 3.3. Identification and Quantification of Active Compound Quercetin

**Figure 5:**
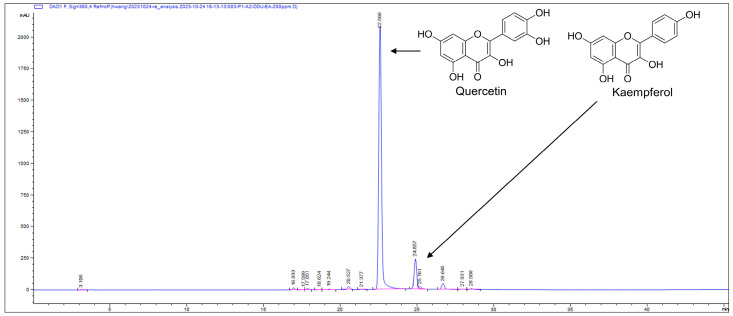
HPLC chromatograms of *A. lobatum* EA extract. Two major peaks indicate quercetin and kaempferol, respectively.

The E80M5 sub-fraction was further purified by prep-HPLC and the sub-fraction was identified as quercetin by NMR (Figure S1). Since the antibiofilm activity of quercetin against *S. aureus* was previously reported [7,22], the current result supports the previous studies. Furthermore, the amounts of quercetin in the *A. lobatum* crude EtOH extract and the EA extract were quantified with HPLC and standard chemicals. The crude EtOH extract contains quercetin at 40.9 ± 0.8 μg/mg and kaempferol at 3.8 ± 0.1 μg/mg (Figure S2), and the EA extract contains quercetin at 391.1 ± 5.4 μg/mg and kaempferol at 30.3 ± 0.3 μg/mg (Figure 5). Additionally, E80M5 contains 615 μg/mg of quercetin and 49 μg/mg of kaempferol (Figure S3). Overall, the amount of quercetin in three extracts is very high. The NMR spectra of kaempferol is shown in Figure S4. The antibiofilm activity of kaempferol, which was weaker than that of quercetin, was also previously reported [23]. These findings unequivocally demonstrate that the substantial presence of quercetin in the *A. lobatum* extract leads to the inhibition of *S. aureus* biofilm formation. Moreover, the concentration of quercetin in these extracts and fractions closely correlates with their antibiofilm activity.

To confirm the antibiofilm activity of quercetin, the effects of quercetin on the biofilm formation of four *S. aureus* strains, including two MSSA and two MRSA strains, were investigated. As shown in Figure 3, quercetin dose-dependently inhibited the biofilm formation of all *S. aureus* strains. For example, quercetin at 20 μg/mL inhibited the biofilm formation of all four *S. aureus* strains, including two MRSA strains, by more than 70% (Figure 3C,D).

### 3.4. Microscopic Examination of the Antibiofilm Activity of the A. lobatum Extract against S. aureus

**Figure 6:**
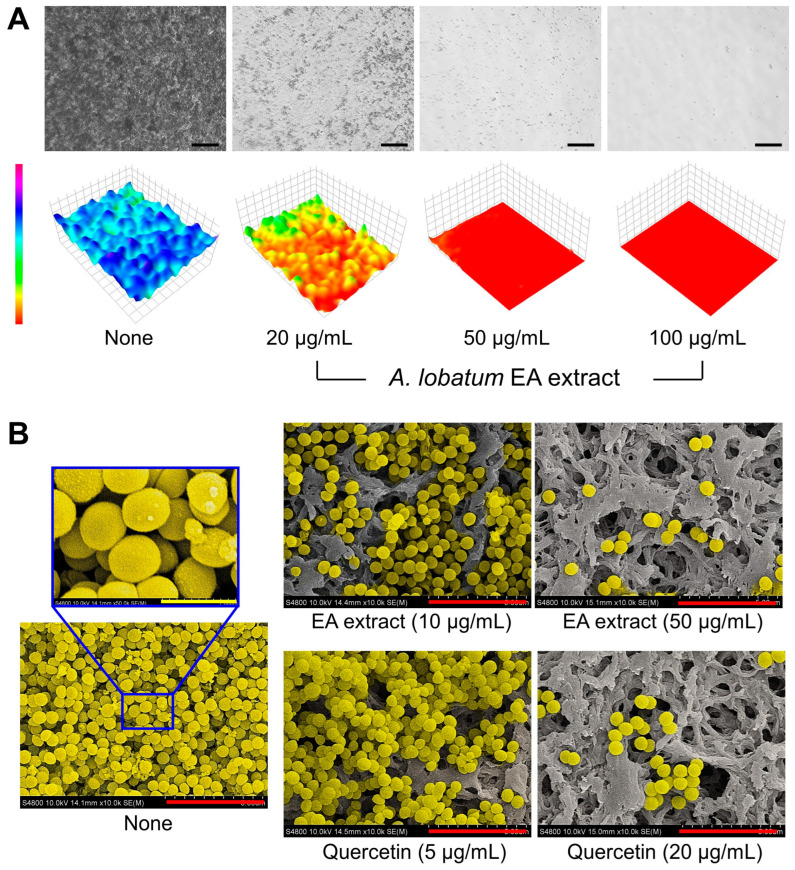
Antibiofilm effects of *A. lobatum* EA extract and quercetin on *S. aureus*. Color-coded 3D images of *S. aureus* ATCC 6538 biofilms in the presence of *A. lobatum* EA extract (**A**), and SEM images in the presence of *A. lobatum* EA extract and quercetin as positive control (**B**). Black, red, and yellow scale bars represent 50, 5, and 1 μm, respectively.

Live imaging microscopy and SEM were used to monitor the reduction of biofilms. Untreated biofilms displayed dense formations, as depicted by blue 3D color images from bright-field microscopy. In contrast, biofilms treated with the *A. lobatum* EA extract ranging from 20 to 100 μg/mL shifted in color from green to red, signifying weak to absent biofilm formation (Figure 6A). SEM analysis further revealed that both the EA extract and quercetin significantly reduced the number of *S. aureus* cells in the biofilms without altering cell morphology (Figure 6B). These findings validate that the *A. lobatum* EA extract at 50 μg/mL and quercetin at 20 μg/mL effectively prevent *S. aureus* biofilm development while preserving cell survival and structure.

### 3.5. Inhibition of Hemolytic Activity by A. lobatum Extract

**Figure 7:**
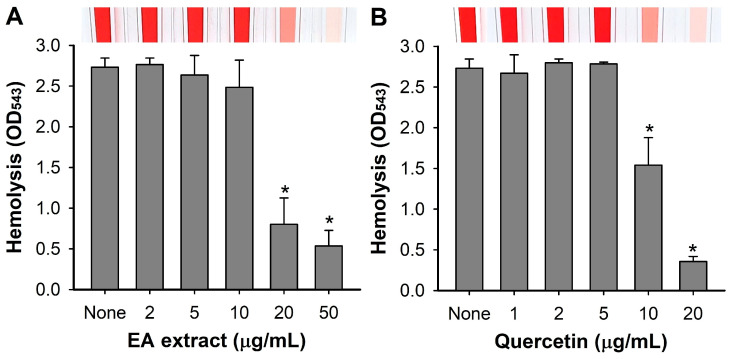
Effects of *A. lobatum* extract and quercetin on hemolytic activity in *S. aureus*. Antihemolytic activity of EA extract (**A**) and quercetin (**B**) against *S. aureus* ATCC 6538. *, *p* < 0.05 vs. non-treated controls (None).

Since *S. aureus* is known to produce hemolysins as a major virulence factor and the antihemolytic activity of quercetin was previously reported [7], the effects of the *A. lobatum* EA extract and quercetin were investigated on hemolytic activity in *S. aureus* 6538. As expected, the EA extract and quercetin progressively diminished the hemolytic activity of *S. aureus* (Figure 7). For example, the EA extract and quercetin at 20 μg/mL inhibited hemolytic activity by 70 and 87%, respectively, which matched with their antibiofilm activities (Figure 3).

## 4. Discussion

The present study highlights the antibiofilm and antihemolytic properties of *A. lobatum* extracts against *S. aureus*, without impacting its planktonic cell growth. Bioassay-guided fractionation of the extract led to the identification of the major active compound quercetin. The comprehensive extract of the whole plant of *A. lobatum* contained quercetin, kaempferol, lobatoside, actinostemmoside, and their glycosides [13]. This study demonstrated that the ethyl acetate extract of *A. lobatum* contains a large amount of antioxidant quercetin and some portion of kaempferol (Figure 5). While the antibiofilm and antihemolytic activities of quercetin against *S. aureus* strains were previously reported [7,23], the current study reports for the first time that the *A. lobatum* extract, rich in quercetin, displayed excellent activities. This indicates the importance of quercetin-rich plants or foods in combating biofilm-related recalcitrant *S. aureus* infections.

The antibiofilm activity of quercetin was previously reported against *S. aureus* strains [7,22,23,24,25,26], *C. albicans* [27], *Escherichia coli* O157:H7, *Vibrio harveyi* [28], and *Pseudomonas aeruginosa* PAO1 [29]. Recent reports have revealed several target proteins of quercetin in *S. aureus* cells. For example, a thermal shift assay showed that quercetin could bind to ClpP (casein hydrolase P) and reduce the thermal stability of ClpP [25]. Molecular docking and kinetic simulation showed that quercetin could bind SarA (a positive biofilm regulator) [22] and ClfB (clumping factor B) [24]. While it is possible that quercetin could bind to several important regulators, more accurate methods are required to validate the real binding to the specific proteins.

The antihemolytic activity of quercetin in *S. aureus* was also previously reported [7,23,30]. Quercetin-rich *A. japonica* extract repressed the intercellular adhesion genes *icaAD*, quorum sensing gene *agrA*, and hemolysin gene *hla* [7]. Also, it was suggested that the antihemolytic activity of quercetin at or near the MIC was related to its effect on the organization of the erythrocyte membrane [30]. However, the exact molecular mechanism of quercetin in *S. aureus* is yet to be unveiled. *S. aureus* encodes α-, β-, γ-, and δ-hemolysins, with the best-studied virulence factor being the pore-forming α-hemolysin, which is encoded by the *hla* gene [2]. At least three global regulators, Agr, Sar, and Sae, coordinately control *hla* expression [31]. The structure of Hla monomer (33.2 kDa) was revealed and the monomer assembles into heptametic complexes [32]. It is interesting to study how quercetin diminishes the expression of *hla* gene or inactivates the Hla protein.

Quercetin widely presents in various foods and plants such as onions, apples, berries, broccoli, kale, leek, and green tea [33] and is a powerful antioxidant to protect plants from various biotic and abiotic stresses [34]. Also, quercetin possesses antibacterial, antiparasitic, and anticancer properties [35]. In this study, the antibiofilm activity of quercetin was validated against four *S. aureus* strains, including two MRSA strains (Figure 3). Microscopic analysis further confirmed that *A. lobatum* extracts effectively curtailed *S. aureus* biofilm formation while preserving cell viability and morphology (Figure 6). While speculative, plants may utilize quercetin to protect against bacterial colonization or biofilm formation. Consequently, quercetin-rich foods and plants could be utilized to treat or prevent biofilm-related infections by antibiotic-resistant *S. aureus.*

This study highlights the potential of *A. lobatum* extracts as effective antibiofilm and antihemolytic agents against *S. aureus*, including MRSA strains. The identification of quercetin as a major active compound in these extracts further supports their efficacy. These findings underscore the importance of quercetin-rich foods or plants for novel antibiofilm agents, particularly in combating biofilm-related infections caused by antibiotic-resistant pathogens like *S. aureus*.

## Data Availability

The datasets used and/or analyzed during the current study are available from the corresponding author on reasonable request. Data are contained within the article.

## Supplementary Materials

The following supporting information can be downloaded at https://www.mdpi.com/article/10.3390/pharmaceutics16081075/s1: Figure S1: ^1^H and ^13^C NMR spectra of quercetin; Figure S2: HPLC chromatograms of *A. lobatum* ethanol extract; Figure S3: HPLC chromatograms of E80M5; Figure S4: ^1^H and ^13^C NMR spectra of kaempferol.
