# Supplementary material for: Antibiofilm and Antihemolytic Activities of Actinostemma lobatum Extract Rich in Quercetin against Staphylococcus aureus
Source: Pharmaceutics. 2024 Aug 16;16(8):1075. doi: 10.3390/pharmaceutics16081075 (PMC11359957; doi:10.3390/pharmaceutics16081075)
Supplement: Supplementary file 1 [file pharmaceutics-16-01075-s001.zip › pharmaceutics-3135301-supplementary.pdf]

Antibiofilm and antihemolytic activities of *Actinostemma lobatum* extract rich in quercetin against *Staphylococcus aureus*

Jin-Hyung Lee[a,1], Yong-Guy Kim[a,1], Ji-Su Choi[b], Yong Tae Jeong[b], Buyng Su Hwang[b,*], Jintae Lee[1,*]

[a]*School of Chemical Engineering, Yeungnam University, 280 Daehak-Ro, Gyeongsan 38541, Republic of Korea*

[b]*Nakdonggang National Institute of Biological Resources, 137, Donam 2-gil, Sangju-si, Gyeongsang-buk-do 37242, Republic of Korea*

[1]These authors contributed equally to this work.

*Corresponding authors.

E-mail addresses: hwang1531@nnibr.re.kr (B.S. Hwang),

jtlee@ynu.ac.kr (J. Lee), Tel: +82-53-810-2533, Fax: +82-53-810-4631

Running Title: Antibiofilm activity of quercetin-rich *Actinostemma lobatum* extract

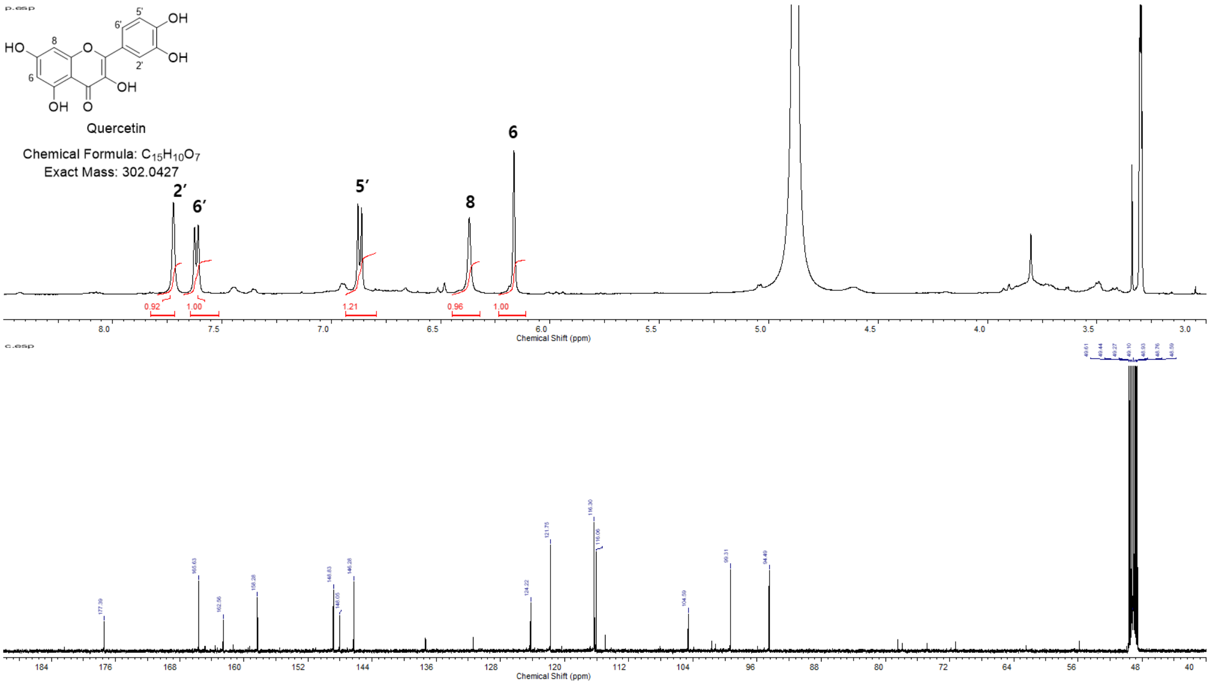

Figure S1. ^{1}H and ^{13}C NMR spectra of quercetin.

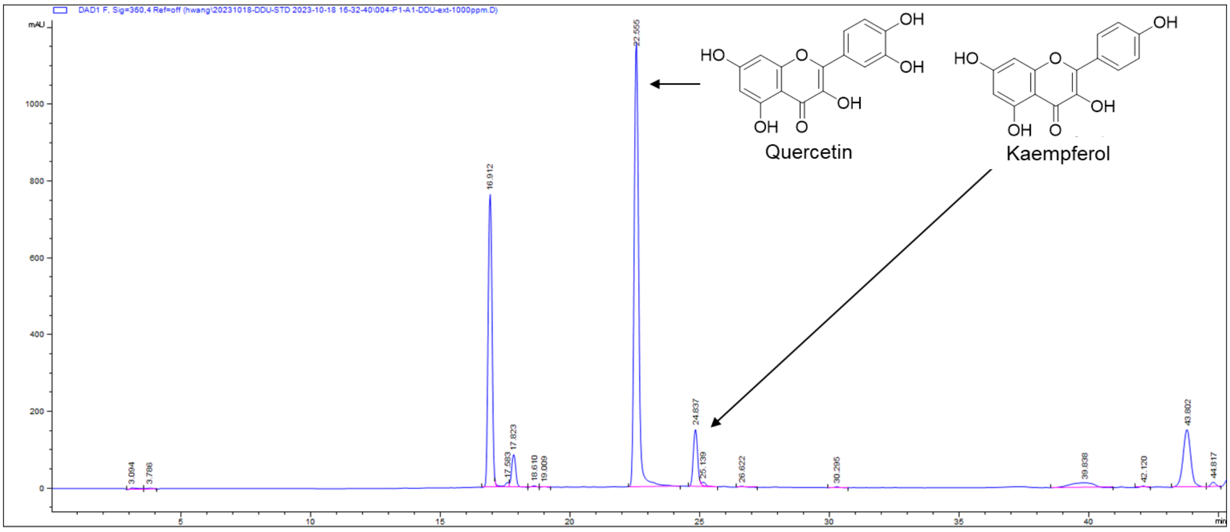

Figure S2. HPLC chromatograms of *A. lobatum* ethanol extract. Two major peaks indicate quercetin and kaempferol.

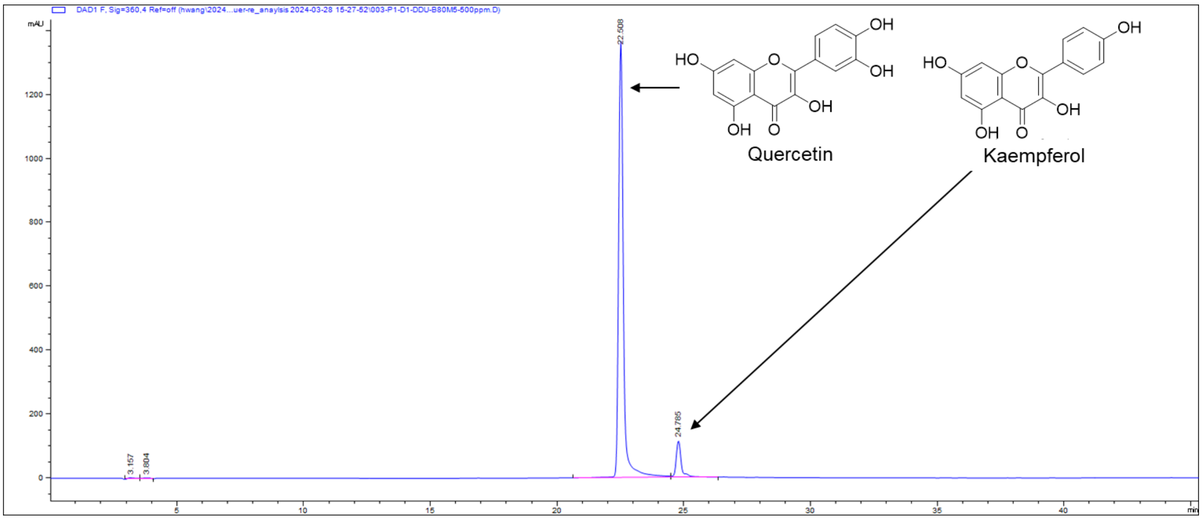

Figure S3. HPLC chromatograms of E80M5. The major peaks indicate quercetin and kaempferol.

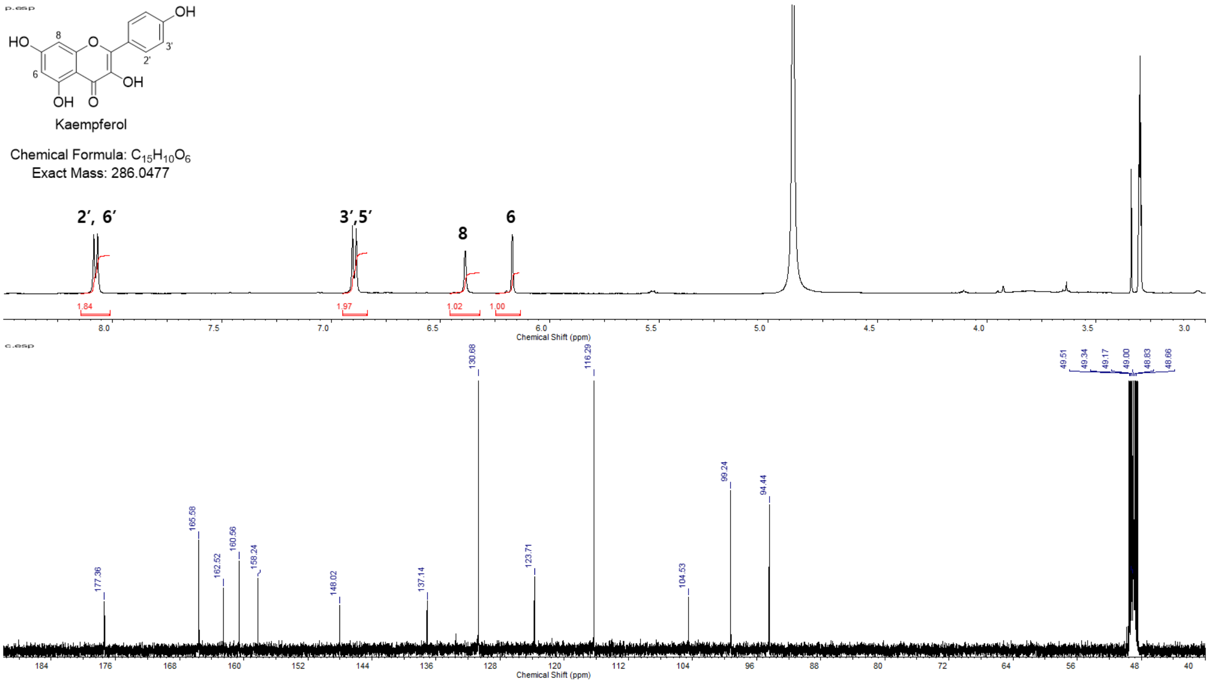

Figure S4. ^{1}H and ^{13}C NMR spectra of kaempferol.
